# Social reward among juvenile mice

**DOI:** 10.1111/j.1601-183X.2006.00295.x

**Published:** 2007-10

**Authors:** J B Panksepp, G P Lahvis

**Affiliations:** † Neuroscience Training Program; ‡ Department of Surgery and Waisman Center for Developmental Disabilities, University of Wisconsin – Madison WI, USA

**Keywords:** Altruism, Co-operation, Emotion, Mouse genetics, *Mus musculus*, Social approach, Social behavior, Sociability, Sociality

## Abstract

Mammalian social relationships, such as mother–offspring attachments and pair bonds, can directly affect reproductive output. However, conspecifics approach one another in a comparatively broad range of contexts, so conceivably there are motivations for social congregation other than those underlying reproduction, parental care or territoriality. Here, we show that reward mediated by social contact is a fundamental aspect of juvenile mouse sociality. Employing a novel social conditioned place preference (SCPP) procedure, we demonstrate that social proximity is rewarding for juvenile mice from three inbred strains (A/J, C57BL/6J and DBA/2J), while mice from a fourth strain (BALB/cJ) are much less responsive to social contact. Importantly, this strain-dependent difference was not related to phenotypic variability in exploratory behavior or contextual learning nor influenced by the genetic background associated with maternal care or social conditioning. Furthermore, the SCPP phenotype was expressed early in development (postnatal day 25) and did not require a specific sex composition within the conditioning group. Finally, SCPP responses resulted from an interaction between two specifiable processes: one component of the interaction facilitated approach toward environments that were associated with social salience, whereas a second component mediated avoidance of environmental cues that predicted social isolation. We have thus identified a genetically prescribed process that can attribute value onto conditions predicting a general form of social contact. To our knowledge, this is the first definitive evidence to show that genetic variation can influence a form of social valuation not directly related to a reproductive behavior.

A breadth of social behavior takes place outside of the highly specific contexts that underlie monogamous pair bonds, mother–infant attachments and territoriality. Approach toward a conspecific, also referred to as social approach, is perhaps the most basic behavioral component of all social interactions, thereby providing a means through which specific relationships between individuals can be established, maintained and strengthened. In some situations, it is conceivable that social approach would be associated with a specific functional outcome (e.g. reproduction during the mating season), while in others the initial motivation to approach a conspecific may be independent of a specific benefit. Under the latter conditions, the likelihood and extent of social approach may thus constrain social experiences that occur only once a certain degree of spatial or temporal proximity exists between individuals. A critical biological problem, then, entails determining whether there are mechanisms and motivations in animals that can support social approach across a variety of situations, irrespective of the ensuing social context.

Classical theories of motivated behavior underscore a role for reward and punishment in essentially all forms of behavioral approach and withdrawal (Glickman & Schiff 1967; Schneirla 1959; Young 1959). Although reward is not necessary for social behavior to occur, reward processes have nevertheless been shown in social contexts that include mating (Agmo & Gomez 1993; Drewett 1973), monogamous pair-bonding (Young & Wang 2004), aggression (Fish *et al.* 2005), maternal–infant attachment (Insel 2003; Lee *et al.* 1999) and rough-and-tumble play among juveniles (Calcagnetti & Schechter 1992; Ikemoto & Panksepp 1992). However, many of these previous studies focused on very specific types of social behavior in relation to ‘control’ conditions (e.g. estradiol-primed vs. nonestrus females, rough-and-tumble play vs. interactions with a partner rendered unresponsive to play solicitations) where a preponderance of social approach behaviors still occurs (Deak & Panksepp 2006; Edwards *et al.* 1990; Pellis & McKenna 1995; Thor & Holloway 1983). Therefore, although specific types of social behavior can be rewarding, it remains unclear whether more general aspects of social behavior, such as social approach or social proximity, also impart a reward value. In this respect, juvenile mice may be an ideal model for assessing the reward value of social interactions within contexts that are not directly related to sexual reproduction, parenting and aggression. For example, juvenile mice express a robust motivation to approach conspecifics (Brodkin *et al.* 2004; Moy *et al.* 2004, Moy 2006; Terranova *et al.* 1993), but their social interactions are rarely sexual or aggressive in nature, and rough-and-tumble play is uncommon (Pellis & Pasztor 1999; Wolff 1981). With the large number of inbred strains that are available (Bucan & Abel 2002; Crawley *et al.* 1997), social interactions among juvenile mice may offer a unique approach to studying the genetic substrates underlying the most basic aspects of sociality.

Here, we employ an experimental model of social reward using a conditioned place-preference (CPP) procedure designed for juvenile mice. Tests of CPP have been used to identify rewarding aspects of aggression (Martinez *et al.* 1995), play (Calcagnetti & Schechter 1992; Douglas *et al.* 2004), sexual interactions (Camacho *et al.* 2004; Jenkins & Becker 2003) and mother–infant bonding (Mattson *et al.* 2001; Weller *et al.* 2001) in various rodent species. During a standard CPP experiment, a series of conditioning sessions occurs within two distinct environmental contexts. One environment is conditioned by the presentation of a reward (traditionally referred to as the unconditioned stimulus, UCS), whereas within a second environment the reward is withheld. Through repeated association, one environment increasingly gains motivational salience and eventually elicits approach behavior comparable with presentation of the reward itself. Upon completion of conditioning, CPP is measured via the expression of an animal’s choice behavior (i.e. time spent in the conditioned environment), even though the reward is not provided during the testing phase of the experiment. Following general principles of Pavlovian conditioning (Maier & Schneirla 1942) and incentive learning (Dickinson & Balleine 2002), CPP tests have an extensive underlying theoretical framework (Bardo & Bevins 2000; Tzschentke 1998) that is consistent with the processes by which neutral stimuli acquire incentive salience (Berridge & Robinson 1998, Berridge 2003). Furthermore, place-conditioning methodologies can be sensitive to the effects of rewarding and aversive UCS-neutral environment pairings. Place-conditioning tests thus offer a general, yet powerful, methodology for inferring an animal’s affective state, irrespective of the direct impact of the UCS. In the present study, we have identified and characterized a genetically specified reward process that appears to facilitate social contact among juvenile mice.

## Materials and methods

### Mouse husbandry

Mice from the A/J (A), BALB/cJ (BALB), C57BL/6J (B6) and DBA/2J (DBA) strains were maintained specific-pathogen free at constant temperature (21 ± 1°C) and humidity (range, 50–60%) under a reversed light cycle (14:10-h light/dark, ‘lights off’ from 1130 to 2130). Mice were housed in standard polyurethane cages (290 × 180 × 130 mm) containing 1/8″ grain-size corn cob bedding (The Andersons, Maumee, OH, USA) and nesting material (Ancare Corporation, Bellmore, NY, USA), with *ad libitum* access to food (Teklad Rodent Diet, Harlan, Madison, WI, USA) and water. Pregnant females were isolated and pups were weaned at postnatal day (PD) 20–21 (day of birth = PD 0). Mice (Jackson Laboratories, Bar Harbor, ME, USA) were routinely introduced to the breeding colony and brother–sister matings were avoided. All experiments were conducted in strict accordance with the guidelines set forth by the institutional care and use committee at the University of Wisconsin – Madison and the national institutes of health (NIH) *Guide for the Care and Use of Laboratory Animals*. All animal husbandry was performed by our own laboratory personnel to maintain gentle and consistent handling of mice.

### General CPP methodology

**Figure 1:**
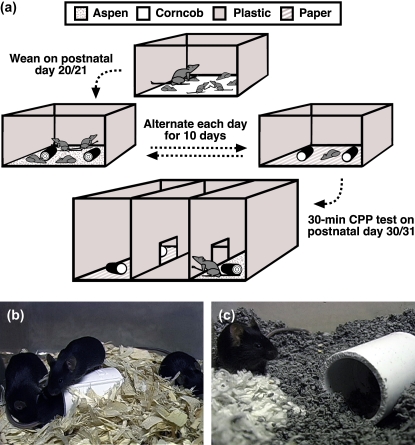
**Conditioning procedure for evaluating the social preferences of juvenile mice.** (a) Following 24 h of social housing in a distinct home cage environment, mice were socially isolated in a second novel home cage environment for the next 24 h. The conditioning context (social or isolate housing) was always counterbalanced relative to its pairing with the home cage environment (aspen or paper bedding). Mice were alternately housed in each environment every 24 h for a total of 10 days. Following the last day of conditioning, which entailed 24 h of social isolation, the spatial location and locomotor activity of individual mice were monitored during a 30-min test session. (b) Photograph of socially housed juvenile B6 mice in an aspen environment. (c) Photograph of an isolated juvenile B6 mouse housed in a paper environment.

We developed a CPP procedure to determine whether social contact among juvenile mice could elicit behavioral approach toward an otherwise neutral environment (Fig. 1a). For the first 24 h following weaning, four mice (two per gender) were housed together in a standard home cage that contained a set of novel environmental cues (social housing condition; Fig. 1b). For the next 24-h period, mice were socially isolated within a second, distinct home cage environment (isolate-housing condition; Fig. 1c). One conditioning environment (‘paper’) included pelleted paper bedding (Cellu-Dri Soft, Shepherd Specialty Papers, Kalamazoo, MI, USA), two schedule 40 1″ polyvinylchloride (PVC) couplers and nesting material. The second conditioning environment (‘aspen’) included aspen shavings (Nepco, Northeastern Products Corporation, Warrensburg, NY, USA), two schedule 40 1″ PVC threaded couplers and nesting material. These two sets of environmental cues were always counterbalanced with respect to their association with the conditioning contexts, and clean bedding, nesting material and PVC tubes were provided at the beginning of each conditioning session. Thus, every 24 h the conditioning procedure entailed a predictable alternation of the home cage living situation with respect to its social and nonsocial stimulus characteristics. Following a number of preliminary studies, we adopted to use conditioning sessions of a 24-h duration because they produced a strong social conditioning response in juvenile mice. In contrast, the use of shorter social conditioning sessions (e.g. 30–60 min within a single compartment of the CPP test arena) generally resulted in smaller and more variable conditioning effects. The decision not to use shorter conditioning sessions also eliminated the undesirable and potentially complicating factors that could have resulted from maintaining mice in continuous isolate housing outside of the conditioning environments.

Following the completion of 10 24-h conditioning sessions, CPP responses of individual (PD 30/31) mice were evaluated in a testing arena (ABS Plastics, Midland Plastic Inc., New Berlin, WI, USA) that allowed mice free movement between three compartments (300 × 150 × 150 mm per compartment) via an opening (50 × 50 mm) in each delimiting wall. With no conditioning cues present, individual mice were familiarized to the testing arena for a 20-min session on each of the 2 days preceding CPP testing. During a 30-min test session, conditioning cues consisting of fresh, unsoiled bedding (20 mm depth) and two clean PVC tubes were provided in the peripheral compartments of the arena, while the central compartment, which did not contain conditioned cues (Lexan® floor), served as a putative neutral environment. Individual mice were placed in the central compartment at the beginning of the habituation and testing sessions, and the arena was then covered with a transparent Plexiglas® top. Tests were videotaped (Sony, DCR-VX2100, Japan) and stored on a Dell Pentium IV PC for subsequent analysis. All CPP tests and habituations were conducted during the dark phase (1300–1900), under dim red illumination. Mouse cages were transported approximately 5 meters to the procedure room >30 min prior to testing.

### Experimental design

#### Experiment 1 – Baseline preferences for the novel environments (i.e. no conditioning)

To evaluate whether juvenile mice developed a preference for the aspen or paper environment in the absence of associated conditioning contingencies, mice were handled according to the CPP procedure described above, except they remained together in a social group as they were alternated daily between the aspen and paper home cage environments. The environment on the first day of conditioning was counterbalanced across all groups to determine whether environmental preferences of mice were sensitive to the environment that had been experienced on the day prior to place-preference testing (i.e. environment preferences for mice tested following 24 h in aspen were compared with those of mice that had spent 24 h in paper prior to testing). For these baseline environmental preference measurements, juvenile mice from the A, BALB, B6 and DBA strains were examined (*N*= 32 mice from seven to eight litters per strain).

#### Experiment 2 – Social conditioning

This experiment addressed whether juvenile mice preferred environments that were associated with living in a mixed-gender social group of littermates vs. social isolation. The conditioning procedure described in the *General CPP Methodology* section was followed (see above). Mice always began the conditioning phase of the experiment in a social group, making social isolation the default housing condition for the 24-h period prior to social conditioned place preference (SCPP) testing. This particular order of conditioning sessions was chosen based on the hypothesis that expression of SCPP responses would be stronger following a period of social deprivation (also see *Experiment 8*). For these measurements of social preference, juvenile mice from the A, BALB, B6 and DBA strains were tested (*N*= 32–36 mice from seven to nine litters per strain). Litter size, sex bias within each litter and maternal experience (primiparous vs. multiparous) were noted and their relationship to SCPP responses was also assessed.

#### Experiment 3 – Food conditioning

This experiment was designed to control for the possibility that strain-dependent differences in SCPP (see *Experiment 2*) were attributable to a more general difference in the ability of juvenile mice to establish a contextual association between the home cage environment and a UCS. Following the conditioning protocol used for Experiment 2, groups of mice were alternated every 24 h between an environment that was paired with *ad libitum* access to standard lab chow and an environment paired with complete food deprivation. Mice began the conditioning phase of the experiment in the food-paired environment, so that CPP testing always occurred after 24 h of food deprivation. For these measurements of food preference, juvenile mice from the A, BALB, B6 and DBA strains were tested (*N*= 24–28 mice from six to seven litters per strain). Weights for all food-deprived mice were monitored and compared with the weights of mice that were maintained under free-feeding conditions during Experiments 1 and 2.

#### Experiment 4 – Social conditioning following cross-fostering

The relationship between the preweaning social environment and SCPP responses was evaluated by conducting a reciprocal cross-fostering experiment with the strains that were the least (BALB) and most (B6) responsive to social conditioning during Experiment 2 (see *Results*). Pups from one strain were replaced with a group of pups from the alternate strain within 12 h of birth. Subsequent mouse husbandry and experimental treatment followed the description above and the conditioning protocol used for Experiment 2. Mice from the BALB and B6 strains were tested (*N*= 16 mice from three to four litters per strain).

#### Experiment 5 – Social conditioning within mixed-strain social cohorts

This experiment tested the possibility that strain differences in SCPP were associated with the collective genetic background of the social conditioning group, rather than the genetic background of individual test mice. Juvenile mice were conditioned in social groups that contained one male and female from the BALB strain, as well as one male and female from the B6 strain. Thus, unlike same-strain social housing (see *Experiments 2* and *4*), the phenotypic variability of juvenile BALB and B6 mice was represented within each social group. In all cases, the social group contained individuals from different litters. Following the conditioning protocol of Experiment 2, a total of 10 mixed-BALB/B6 social groups were examined (*N*= 20 mice from 10 litters per strain).

#### Experiment 6 – Social conditioning within same-gender social cohorts

This experiment addressed whether the acquisition of SCPP responses by juvenile B6 mice was influenced by a differential preference for conspecifics of the opposite sex. Mice were socially housed in all-male or all-female groups that contained four individuals from the same litter, and were conditioned following the procedure described for Experiment 2. A total of eight social groups were tested (*N*= 16 mice from three to four litters per gender).

#### Experiment 7 – Social conditioning of younger mice with an abbreviated conditioning protocol

To further evaluate whether the social conditioning response reflected a differential preference for individuals of the opposite sex, expression of SCPP was examined in juvenile B6 mice (PD 25/26) that do not exhibit behavioral indications of sexual interest (Panksepp, J.B. *et al.*, under review). At weaning (PD 20/21), two males and two females from the same litter were formed into a social group and housed for the next 24 h within a standard home cage environment (with corn cob bedding and nesting material). The next day, mice were given a preconditioning test to confirm a lack of preference for the aspen or paper environments prior to conditioning. The measurement of environment preferences at this particular time-point was therefore analogous to the baseline measurements taken for the B6 strain during Experiment 1, except that mice were responding to the environmental cues when they were still completely novel (unlike B6 mice in Experiment 1, at testing the mice in this experiment had not yet experienced the aspen or paper environments in the home cage). Following the preconditioning test, mice were placed back into a social housing context within one of the two novel home cage environments (environment-housing pairings were determined randomly, but counterbalanced across the groups). Over a total of 4 days (two social housing and two isolate-housing conditioning sessions) conditioning proceeded as described for Experiment 2. Each mouse was familiarized to the test environment for 20 min on the 2 days preceding testing and a 30-min SCPP test was conducted following 24 h in the isolate-housing conditioning (postconditioning test). Six mixed-gender social groups were tested (*N*= 24 mice from three litters).

#### Experiment 8 – Social conditioning responses following 24 h of social housing

To assess whether social isolation was a necessary precondition for the SCPP phenotype to be expressed, juvenile B6 mice were subjected to the social conditioning procedure used for Experiment 2 with the order of conditioning sessions (social or isolate housing) reversed. Thus, for this experiment juvenile mice were weaned into the isolate-housing context for the first 24 h of conditioning and were tested for SCPP after 24 h of social housing. Six mixed-gender social groups were tested (*N*= 24 mice from six litters).

#### Experiment 9 – Contribution of social approach and isolation avoidance to social conditioning responses

To determine whether there were independent (social) approach and (isolation) withdrawal components underlying the SCPP phenotype, the conditioning protocol of Experiment 2 was modified to include a third environment of shredded newspaper with lemon-scent (‘newspaper’ environment). Lemon extract (500 μl) was added immediately before mice were introduced to the home cage for a conditioning session.

For one group of juvenile B6 mice (*N*= 32 mice from six litters), individuals were socially conditioned within one home cage environment (pairings with aspen and paper were counterbalanced across the social groups) and isolate housed within the newspaper environment. Following the final 24 h of social isolation, mice were tested with the aspen and paper environments present in the peripheral compartments of the testing arena to assess whether they expressed a preference for the socially paired environment vs. a completely novel environment.

The extent to which withdrawal from social isolation contributed to the SCPP response of juvenile mice was evaluated in individuals that were socially isolated within the aspen or paper environment, and socially housed within the newspaper environment during conditioning. For these mice (*N*= 32 mice from six litters), CPP was evaluated following 24 h of social isolation with the aspen and paper environments (i.e. an isolation paired environment vs. a completely novel environment) present in the peripheral compartments of the testing arena.

To control for the potential effects of presenting a novel set of environmental cues in the testing arena, the preferences of a third group of mice (*N*= 32 mice from seven litters) that received 24-h social housing sessions in the aspen or paper environment alternated with social housing in the newspaper environment were assessed. During testing, these mice were presented with the aspen and paper in the testing arena (i.e. a completely novel environment vs. a familiar environment), environments for which there could have been no explicit relationship to the social housing context.

### Behavioral measurements

Video files of each CPP test were scored using computer-assisted analysis software (ButtonBox 5.0, Behavioral Research Solutions, Madison, WI, USA). Twenty-five percent of all CPP tests (149 of 596 mice) were scored in duplicate and interrater reliabilities were high for each experiment (Pearson’s correlation > 0.99 for each test). Preference scores were generated by calculating the time spent by each mouse in the rewarded (social or food) environment *minus* the time spent by each mouse in the reward-impoverished (isolate or food deprivation) environment. For mice that did not receive conditioning in Experiments 1 and 7, baseline preference scores were arbitrarily calculated as the time spent by each mouse in the aspen environment *minus* the time spent by each mouse in the paper environment. Compartment entries were measured as the total number of crosses into any of the three compartments within the testing arena.

In several instances, mice were noticeably inactive during the place-preference tests. We categorized these mice as ‘unresponsive’ if they entered one peripheral compartment of the testing arena and remained there for at least the first 20 min of the 30-min test session. Overall, these unresponsive mice made <5 entries into each compartment of the testing arena and constituted <9% of mice that were tested in experiments in which all four strains were sampled (Experiments 1–3). Because their relative preference for the conditioning environments could not be assessed, unresponsive individuals were not included in any of the statistical comparisons. However, it was noteworthy that a majority of the unresponsive mice were from groups that did not receive conditioning (see Results), which provided us with an additional way to measure the effects of conditioning on behavioral responsiveness.

### Statistical analysis

For Experiments 1–5, two-way analyses of variance (anovas) with strain and gender as between-group factors were used to evaluate preference scores and compartment entries. Subsequent *post hoc* comparisons were carried out with Tukey’s honestly significant difference (HSD) tests or orthogonal contrasts that were nested within the between-groups variance estimate (Sokal & Rohlf 1995). The relationship between SCPP responses (from Experiment 2), and litter size and gender bias was analyzed with Pearson’s product–moment correlations, while a strain-dependent influence of maternal experience on SCPP was assessed with a two-way anova. For Experiments 6 and 7, one-way anovas (with repeated measures for the pre- vs. post-conditioning comparison of Experiment 7) were used to evaluate gender differences on the expression of SCPP, and for Experiments 6 and 8 two-tailed *t*-tests were employed to assess whether there was an overall SCPP for each group (H_Ø_= 0). A two-way anova with gender and conditioning group as between-group factors was used to evaluate preference scores for Experiment 9, with follow-up orthogonal contrasts to make specific comparisons between groups. For all statistical tests, *P* < 0.05 was considered significant.

## Results

### Strain-dependent variation

**Figure 2:**
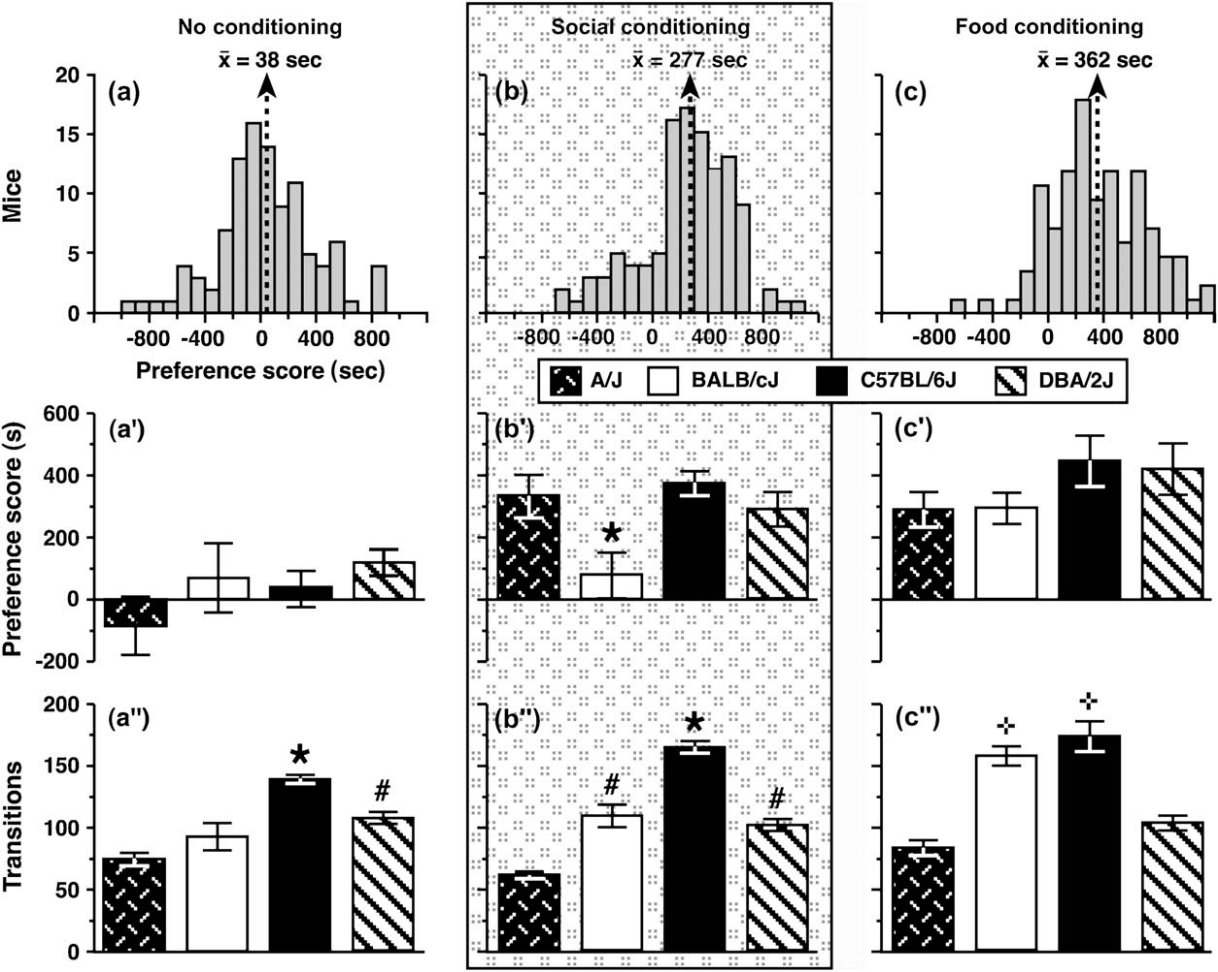
**Strain-dependent variation in the conditioning responses of juvenile mice.** (a–c) Frequency distributions illustrate the number of mice (ordinate) expressing a particular preference score (abscissa) following (a) no conditioning, (b) social conditioning or (c) food conditioning. Mice from all of the strains were included in the distributions. (a′–c′) Juvenile mice did not differentially approach or explore the environments (a′) without conditioning. (b′) Social conditioning resulted in a CPP for mice from three strains but not BALB mice (Tukey’s HSD tests, *P < 0.05 for all *post hoc* tests comparing BALB with the other strains). (c′) Mice from all of the strains learned the conditioning contingency when food was used as a reward. Preference scores were calculated as the duration spent in the reward-paired (social or food) environment *minus* the duration spent in the reward-impoverished (isolation or food deprivation) environment. (a″–c″) There were strain-dependent differences in locomotor activity (a″) without conditioning, (b″) with social conditioning and (c″) with food conditioning. There was no difference in exploratory activity between BALB and B6 mice that were tested following the food conditioning procedure (Tukey’s HSD tests, *P < 0.05 compared with all other strains, ^#^*P* < 0.05 compared with the A strain, ^+^*P* < 0.05 compared with the A and DBA strains). Data in panels a′–c′ and a″–c″ are presented as the mean ± SEM.

**Figure 3:**
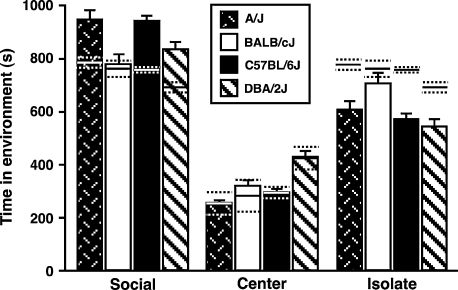
**Spatial distributions of juvenile mice following social conditioning.** Social preference scores of juvenile mice in Fig. 2b′ are replotted as the untransformed spatial data that was taken for each mouse. The continuous (black or white) horizontal line associated with each bar indicates the average time spent in the aspen/paper (peripheral) environments or the central environment by juvenile mice that were not conditioned (see *Experiment 1* and Fig. 2a′). The dashed lines that bound each sample mean denote the 95% confidence interval for the respective measurement. The SCPP data illustrated in bar-form are presented as the mean ± SEM.

Juvenile mice approached and explored environments associated with social contact to a greater degree than environments associated with social isolation (Fig. 2b; *t*= 9.0, df = 112, *P* < 0.0001; see *Experiment 2* in *Materials and methods*). In particular, mice from the A, B6 and DBA strains exhibited a robust SCPP response (Fig. 2b′). By contrast, juvenile BALB mice expressed a substantially lower SCPP response (Fig. 2b′; *F*_3,109_= 4.2, *P*= 0.008), exploring the two conditioning environments to a similar extent (*t*= 1.0, df = 23, *P*= 0.84). Importantly, without prior conditioning, mice from all of the strains lacked a preference for the aspen and paper environments (Fig. 2a and a′; *F*_3,99_= 1.6, *P*= 0.18; see *Experiment 1*). Furthermore, preferences for relative environmental novelty (preference score = time in environment used for conditioning session 10 *minus* time in environment used for conditioning session 9) were not apparent for unconditioned mice from any of the strains (*F*_3,99_= 1.9, *P*= 0.14). Examination of the untransformed place-preference data from Experiments 1 and 2 (Fig. 3) indicated that the SCPP responses of juvenile mice from the A, B6 and DBA strains arose specifically from a differential allocation of time spent in peripheral (experimental) compartments of the testing arena, with no changes in the duration of time mice spent within the central compartment. Although refractory to social conditioning, there did appear to be an overall strain difference in the time that juvenile mice spent exploring the central compartment of the testing arena (see Fig. 3).

Evaluation of gender-specific SCPP responses suggested that social conditioning was absent in BALB females [mean ± standard error of the mean (SEM); preference score = 5 ± 107.3 s] and diminished in BALB males (174 ± 90.8 s), consistent with a trend toward gender differences in the expression of SCPP (*F*_1,111_= 3.6, *P*= 0.06). However, lack of a strain-by-gender interaction (*F*_7,105_= 0.8, *P*= 0.50) indicated that males expressed stronger SCPP responses irrespective of their particular genetic background.

A food conditioning protocol (see *Experiment 3*) was employed to determine whether the strain differences in SCPP were attributable to a more general difference in the ability of juvenile mice to form contextual associations. Mice from all four strains exhibited a strong CPP when the conditioning environments were associated with food availability (Fig. 2c and c′; *F*_3,92_= 1.2, *P*= 0.32) and there was no difference between males and females in this response (*F*_1,94_= 0.4, *P*= 0.54). Juvenile mice that expressed the strongest CPP response to food availability were from strains (B6 and DBA) that lost the most weight as a consequence of the food deprivation schedule used for conditioning (values expressed as percentage ± SEM of the average weight of mice from the same strain in Experiments 1 and 2; A – 73 ± 3.4, BALB – 78 ± 1.7, B6 – 70 ± 2.1, DBA – 70 ± 2.2; *F*_3,95_= 2.7, *P*= 0.04).

A portion of juvenile mice exhibited diminished exploratory behavior during testing. This very low level of exploratory behavior was only observed for juvenile mice from the A and BALB strains, with no difference between males and females. In general, these unresponsive individuals moved throughout one peripheral compartment of the arena but rarely made transitions between compartments of the testing arena. Furthermore, the data points representing these mice constituted a conspicuous peak (near the origin of the abscissa) within an otherwise normal frequency distribution of compartment entries. Of mice that were not conditioned (Experiment 1), 19% of A mice and 50% of BALB mice were categorized as unresponsive because they did not leave the peripheral compartment of the arena which was first entered within 20 min of the beginning of the testing session (see *Materials and methods*). These individuals expressed a mean preference score and number of compartment entries >6.5 standard deviations (SD) and >2 SD from the group averages of unconditioned mice, respectively. For the analysis of SCPP (Experiment 2), 19% of A mice and 28% of BALB mice were similarly unresponsive, expressing deviant place-preference scores and exploratory behavior (>3.5 SD and >2 SD, respectively, as reported above). Of note, all individuals from the food conditioning experiment (Experiment 3) were responsive.

Although unresponsive mice were not included in the statistical comparisons, there still was strain-dependent variation in exploratory activity (Fig. 2a″; *F*_3,99_= 23.7, *P* < 0.0001), with the largest relative difference between the strains reaching ∼1.9-fold. Strain differences in activity for unconditioned control mice were similar to mice that had experienced social conditioning (Fig. 2b″; *F*_3,109_= 63.6, *P* < 0.0001). Importantly, mice from the most active (B6) and least active (A) strains expressed comparable SCPP responses (see Fig. 2b′). There also were strain-dependent differences in locomotor activity when food availability was used as the UCS (Fig. 2c″; *F*_3,92_= 25.1, *P* < 0.0001). For Experiments 1–3, we found no significant gender or strain-by-gender effects on the number of compartment entries made during the testing period. The deprivation protocol used for Experiment 3, however, increased the rate of compartment transitions by BALB mice, such that BALB exploratory behavior was indistinguishable from B6 mice (Fig. 2c″; orthogonal contrast for BALB vs. B6, *F*_1,88_= 1.9, *P*= 0.17).

### Social context

Focusing on the strains that were the most (B6) and least (BALB) responsive to social conditioning, we did not detect a relationship between SCPP responses and litter size (*r*= 0.19 and −0.05 for the BALB and B6 strains, respectively, *P* > 0.05 for both tests), gender ratio within each litter (*r*=−0.17 and 0.02, *P* > 0.05, as reported above) and maternal experience (strain × maternal experience interaction, *F*_1,52_= 2.8, *P*= 0.10).

**Figure 4:**
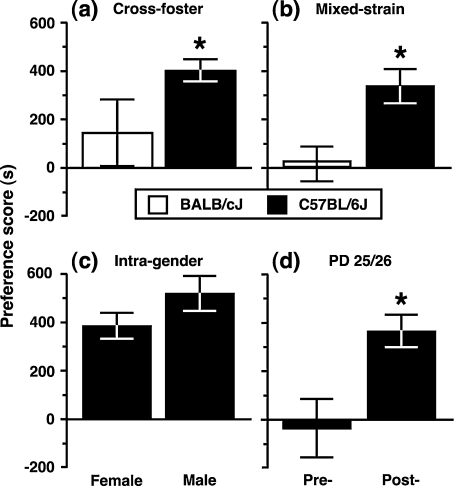
**Variation in social conditioning as a function of maternal care, social group characteristics and age.** (a) When pups were cross-fostered to a mother of the alternate strain within 12 h of birth juvenile B6 mice, but not BALB mice, expressed SCPP (main effect of strain, *P= 0.02). (b) The strain difference in SCPP persisted when BALB and B6 mice (one per gender for each strain) were conditioned together in the same social group (main effect of strain, *P= 0.004). (c) Male and female B6 mice conditioned in same-sex groups also expressed robust SCPP responses. (d) At PD 25/25, SCPP was apparent for juvenile B6 mice following four conditioning sessions (*effect of conditioning, *P*= 0.003). Data in each panel are presented as the mean ± SEM.

To determine whether strain-dependent variation in SCPP was influenced by differences in maternal care, BALB and B6 mouse pups were cross-fostered to mothers of the alternate strain (see *Experiment 4*). Overall, five BALB mice were excluded from the analysis because of inactivity (see *Materials and methods*) and there was a gender difference in the expression of SCPP responses (*F*_1,25_= 9.0, *P*= 0.006), consistent with the previous finding of stronger social conditioning responses by male mice. However, despite differential upbringing, SCPP responses continued to segregate with the genetic background of the juvenile mice (Fig. 4a; *F*_1,25_= 6.1, *P*= 0.02).

Conditioning BALB and B6 juvenile mice within mixed-strain social groups allowed for the relationship between SCPP responses and social group characteristics to be assessed (see *Experiment 5*). Strain-dependent differences in SCPP persisted when BALB and B6 mice were conditioned together in mixed-strain social groups (Fig. 4b; *F*_1,32_= 9.5, *P*= 0.004). Similar to our findings for Experiments 2 and 4, a proportion of BALB mice were excluded from the analysis because of reduced exploratory activity (six mice) and males tended to express stronger SCPP responses relative to females, although this pattern did not reach statistical significance (*F*_1,32_= 2.5, *P*= 0.13). The continued expression of SCPP by juvenile B6 mice housed in mixed-strain housing also shows that development of the social conditioning response was not dependent on shared kinship among group members.

Juvenile B6 mice were conditioned in same-sex social groups (four males or four females per group) to assess whether the social conditioning response was related to a preference for conspecifics of the opposite gender. In this experiment, measures of SCPP were not different between the sexes (*F*_1,23_= 1.4, *P*= 0.24), even though mice from both groups expressed a robust social conditioning response (Fig. 4c; males –*t*= 6.9, df = 15, *P* < 0.0001; females –*t*= 6.8, df = 15, *P* < 0.0001). The influence of sexual interest on the development of SCPP responses was further assessed in juvenile B6 mice that were exposed to an abbreviated conditioning protocol (see *Experiment 7*). At a stage of development when there are not differential behavioral interests for mice of the opposite gender (Panksepp, J.B. *et al.*, under review), juvenile B6 mice expressed a strong SCPP response (Fig. 4d; *F*_1,23_= 10.7, *P*= 0.003).

### Motivational systems

Our results from the previous experiments showed that the SCPP responses of juvenile B6 mice were generally unresponsive to modifications of the social conditioning environment. However, prior social isolation (see *Experiment 8*) was critical for the expression of SCPP by juvenile B6 mice because social conditioning responses were not evident when the order of conditioning sessions was reversed and CPP tests were conducted following 24 h of social housing (preference score = 50 ± 54.6 s; *t*= 0.91, df = 23, *P*= 0.37).

**Figure 5:**
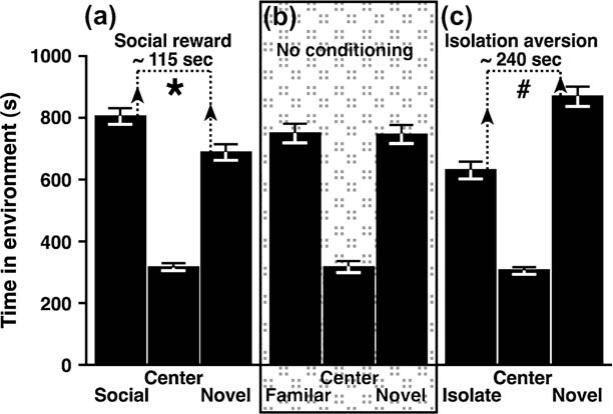
**Social reward and isolation aversion in juvenile B6 mice.** (a) B6 mice differentially approached socially conditioned cues relative to novel cues (*orthogonal contrast for social approach vs. novelty approach, *P*= 0.03, social approach = time in social environment *minus* time in novel environment). (b) Unconditioned B6 mice did not respond differentially to the presentation of novel cues in the testing arena (novelty approach = time in novel environment *minus* time in familiar environment). (c) B6 mice approached novel environments only when the other peripheral compartment of the testing arena contained cues that predicted social isolation (^#^orthogonal contrast for isolation aversion vs. novelty approach, *P* < 0.001, isolation aversion = time in novel environment *minus* time in isolate environment). Data in each panel are presented as the mean ± SEM.

In a final experiment, we used a third conditioning environment to evaluate whether there were distinct contributions of social approach and withdrawal from social isolation to the development of SCPP by juvenile B6 mice (see *Experiment 9*). Following conditioning, preferences for environments that were paired with social housing and social isolation were dissociable (*F*_2,90_= 6.6, *P*= 0.002). In particular, mice specifically approached environments that had been associated with social contact during conditioning (Fig. 5a; orthogonal contrast for social approach vs. novelty groups, *F*_1,90_= 4.7, *P*= 0.03), even when the influence of presenting a novel set of environmental cues in the CPP testing arena was controlled for (Fig. 5b). Juvenile B6 mice also avoided environments that were paired with social isolation, preferring to spend time in the compartment of the test arena that contained a set of completely novel environmental cues (Fig. 5c; orthogonal contrast for isolation avoidance vs. novelty groups, *F*_1,90_= 13.0, *P* < 0.001). These two distinct phenotypes were not gender specific (conditioning × gender interaction, *F*_2,90_= 0.8, *P*= 0.43) and environmental novelty did not directly influence exploratory behavior during testing (see Fig. 5b). When independent measures of social approach (social *minus* novel =∼115 s) and isolation avoidance (novel *minus* isolate =∼240 s) were combined, the resulting value was very similar to the average SCPP score obtained for juvenile B6 mice during Experiment 2 (social *minus* isolate =∼370 s; see Fig. 2b′).

## Discussion

In the present study, we have found that living in a social group can serve as a potent reward for young mice from three inbred strains (A, B6 and DBA) by enabling the assignment of new preferences onto previously neutral environments. However, this SCPP response was greatly reduced in juvenile mice from the BALB strain. Identification of this strain difference adds substantially to previous findings regarding the social approach behaviors of juvenile inbred mouse strains (Brodkin *et al.* 2004; Moy *et al.* 2004, Moy 2006; Sankoorikal *et al.* 2006) because it demonstrates that a value can be assigned solely to the opportunity for social interaction. In general, an association between social approach and reward provides a conceptually powerful mechanism by which approach behaviors can be initiated and maintained (Glickman & Schiff 1967; Kelley 2005; Schneirla 1959; Young 1959). The fundamental importance of psychological concepts such as ‘wanting’, ‘expectancy’ and ‘seeking’ for studying brain reward systems (Berridge & Robinson 1998; Ikemoto & Panksepp 1999; Kalivas & Volkow 2005) illustrates that one basic function of reward systems is to mediate approach behaviors in relation to biologically significant events (Kelley & Berridge 2002). To our knowledge, the present findings are the first to show that the basic framework of reward theory can be applied to social approach among juvenile mice, and that this reward process is influenced by genetic variation.

In contrast to juvenile mice from the A, B6 and DBA strains, which exhibited a robust CPP response for both social contact and food availability, BALB mice only expressed a place preference when environments were conditioned by access to food. A recurrent question during our study focused on whether the reduced preferences of BALB mice after social conditioning were attributable to a more general strain difference in exploratory behavior. For experiments in which all four strains were sampled (Experiments 1–3), approximately 9% of mice were categorized as ‘unresponsive’ during the place-preference tests because they exhibited very low levels of exploratory behavior. Interestingly, all of these unresponsive individuals were of the A or BALB genetic background, strains that have been previously described as expressing high levels of generalized anxiety (Bouwknecht & Paylor 2002; Cohen *et al.* 2001; Moy *et al.* 2006; Priebe *et al.* 2005). From this point-of-view, the greatly diminished number of compartment transitions by some juvenile A and BALB mice could indicate that 24 h of social isolation prior to testing was insufficient to mediate an active exploratory response in a proportion of individuals from each strain. Such an interpretation would be compatible with the idea that anxiety can serve as a constraint on social responsiveness (e.g. see Bielsky *et al.* 2005; Brodkin 2006).

However, the occurrence of unresponsive mice during place-preference testing was additionally informative because it provided us with an independent measure of appetitive motivation following conditioning. Individuals from the unconditioned control experiment (Experiment 1) never experienced a reward contingency in relation to the home cage environments, and many BALB mice (50%) from this group were unresponsive. By contrast, fewer BALB mice (28%) from the socially conditioned group were unresponsive (Experiment 2), while all BALB mice that received food conditioning (Experiment 3) moved readily throughout the place-preference arena upon testing. Thus, juvenile BALB mice appeared to be particularly responsive to food deprivation; the frequency of compartment transitions by BALB mice actually increased to a level indistinguishable from B6 mice that were conditioned with the same UCS. These findings indicate that the responsiveness of juvenile mice during place-preference testing was probably sensitive to the presence of a specific reward contingency or motivational state. Consistent with this interpretation, the occurrence of unresponsive A mice was also reduced to zero following food deprivation.

Our results suggest that food availability was a much more salient conditioning stimulus for juvenile BALB mice compared with social contact, whether we utilize the CPP response the frequency of exploratory transitions or the number of unresponsive mice as a phenotypic end-point. Indeed, following social conditioning, a majority (72%) of juvenile BALB mice surpassed the criterion for behavioral responsiveness, as indicated by compartment transitions during testing. Nevertheless, these BALB mice expressed reduced place-preference responses. By contrast, juvenile A mice made the fewest number of compartment transitions of all of the strains but exhibited strong CPP responses both for social contact and food availability. Furthermore, we did not detect an association between the number of compartment entries and the magnitude of SCPP responses for mice that met the criterion for behavioral responsiveness during testing (data not shown). Although additional experiments are necessary to clarify the precise relationship between unresponsive mice and the social conditioning response, our present findings are consistent with the interpretation that a specific social-motivational process influences strain-dependent variation in SCPP. Because a relationship between SCPP and strain differences in exploratory behavior, contextual learning, maternal care or the phenotypic characteristics of mice used as the UCS during social housing was not observed, we hypothesize that the social conditioning responses of juvenile mice are moderated by a reward process that can assign value onto social contact.

The finding that expression of the SCPP phenotype by juvenile B6 mice was not dependent on the gender identity of their respective social partners leads to the intriguing possibility that reward mediated by social contact can occur in the absence of a direct opportunity to increase reproductive output. Most previous demonstrations of social reward have been associated with a specific opportunity to gain reproductive benefits (e.g. pair-bonding, mating opportunities, mother–infant attachment or social dominance). However, an exception to this is the strong motivation for social play among adolescent (PD 30–40) rats, where reward strength is positively associated with the degree to which individuals engage in rough-and-tumble play bouts (Calcagnetti & Schechter 1992; Deak & Panksepp 2006; Thor & Holloway 1983). In rats, play behavior is expressed more strongly by males (Olioff & Stewart 1978), exhibits sensitivity to perinatal hormones (Meaney *et al.* 1983) and ultimately promotes the acquisition of behavioral skills that may be beneficial later in adulthood (Panksepp 1981; Van den Berg *et al.* 1999a, Van den Berg 1999b). In contrast to rats, juvenile mice rarely engage in patent forms of social play (Pellis & Pasztor 1999; Wolff 1981). Adolescent development entails bodily, cognitive and emotional changes (Sisk & Foster 2004; Spear 2000) that usually co-occur with major transitions in social relationships (Douglas *et al.* 2003; Smetana *et al.* 2006). Among adolescent mice, increased novelty seeking and risk-taking behaviors (Laviola & Terranova 1998) may be adaptive as they disperse from their natal home range (Byrom & Krebs 1999; Koopman *et al.* 2000; Nelson & Mech 1984). Adolescence is also marked by changes in social dynamics (Ebensberger 2001; Gerlach 1998) including a tendency to form dyadic groupings during dispersal (Drickamer *et al.* 2003). In this context, a general social reward process might serve to maintain social contact during the transient period of adolescent development.

Alternatively, juvenile social reward could reflect an uncommitted or undifferentiated state of social responsiveness that becomes specialized only when new reproductive and survival challenges arise during adulthood. In this respect, several recent studies have suggested that social experiences are intrinsically valuable even when there is no clear opportunity for the approaching individual to benefit. For instance, although submissive male mice withdraw from unfamiliar males (Berton *et al.* 2006), subordinate mice express a strong preference for contact with a familiar dominant counterpart as long as a barrier limits the occurrence of agonistic interactions (Van Loo *et al.* 2001). Similarly, subordinate mice approach and prefer sensory cues that have been conditioned by scents of dominant mice (Fitchett *et al.* 2006). Adult female mice will work vigorously for social contact with others, regardless of whether the ensuing social interaction becomes affiliative, antagonistic or sexual in nature (Matthews *et al.* 2005). Taken together, these studies support the possibility that social contact can be inherently rewarding, irrespective of outcome.

In the last experiment, we found that the expression of SCPP by juvenile B6 mice was moderated by at least two distinct motivational processes. One process we term *social reward*, as B6 mice selectively preferred environments that had been paired with social housing even when cues that predict social isolation were not available. Conversely, we refer to the second process as *isolation aversion* because B6 mice preferred completely novel environments only when cues that predict social isolation were presented in the testing arena. Importantly, the SCPP response was evident only when mice had experienced a certain level of social isolation prior to testing. The expression of SCPP by juvenile mice thus complements studies that have addressed how motivational processes interact and ultimately how they come to modulate behavioral responsiveness (Berridge & Robinson 1998; Broekman *et al.* 1988; Deaner *et al.* 2005; Dickinson & Balleine 2002; Ikemoto & Panksepp 1992; Jenkins & Becker 2003; Koch & Peters 1987; Pettijohn 1981; Schuster & Perelberg 2004). From a psychobiological perspective, social reward and isolation aversion are consistent with the positive affective experiences that occur during social reunion and the negative affective states that accompany social exclusion, respectively (Depue & Morrone-Strupinsky 2005; Panksepp 1998). From a genetic perspective, these processes could be studied as distinct endophenotypes, each with a specifiable genetic substrate and contribution to an individual’s level of sociability. In the future, it will be particularly interesting to address whether the diminished SCPP responses of juvenile BALB mice result selectively from a reduction in social reward *or* isolation aversion. Although we did not conduct such an experiment in the present study, the strain difference in SCPP appears to have arisen from strain-dependent variation in both processes (see Fig. 3).

In their classic paper, Glickman & Schiff (1967) hypothesized that the ability to ‘initiate, guide and maintain’ (pp. 83) behavioral approach toward a goal was the most defining characteristic of a reward. To the extent this assertion can be generalized, a reward should then facilitate behavioral approach irrespective of its co-occurrence with other aspects of the environment. Thus, it is also intriguing to consider whether the strain difference in SCPP identified here could be related to the differences that have been described for juvenile mice that were given direct access to a conspecific (Brodkin *et al.* 2004; Moy *et al.* 2006). Although the present experiments were conducted with mice that were raised in the laboratory, in principle a motivation for social contact driven by a reward process could have broader implications. A close degree of spatial proximity between individuals can either be a prerequisite to, or an outcome of, several distinct patterns of social interaction that are not directly associated with the opportunity for sex (Dugatkin 2002; Hamilton 1971; Roberts 2004; Schuster & Perelberg 2004; Trivers 1971; Wilson 2004). To our knowledge, the present demonstration of social reward provides the first evidence that genetic factors can influence the extent to which an animal values nonreproductive, social interactions.
